# Identification of an isoflavone 6-hydroxylase involved in tectorigenin biosynthesis in kudzu (*Pueraria montana* var. *lobata*) and the efficient production of 6-methoxyisoflavones

**DOI:** 10.5511/plantbiotechnology.26.0330b

**Published:** 2026-06-25

**Authors:** Kai Uchida, Masami Yokota Hirai

**Affiliations:** 1 RIKEN Center for Sustainable Resource Science, 1-7-22 Suehiro-cho, Tsurumi-ku, Yokohama, Kanagawa 230-0045, Japan

**Keywords:** CYP76F318, Isoflavone 6-hydroxylase, *Pueraria montana* var. *lobata*, tectorigenin

## Abstract

Kudzu (*Pueraria montana* var. *lobata*) produces several isoflavones that have beneficial effects in humans. These isoflavones accumulate in a tissue-specific manner, with 5,7,4′-trihydroxy-6-methoxyisoflavone (tectorigenin) and its derivatives accumulating mainly in flowers. Tectorigenin has various beneficial effects on humans and is in high demand, but its biosynthesis has not been elucidated, making large-scale production difficult. In this study, we elucidated the steps in tectorigenin biosynthesis in kudzu by identifying a novel isoflavone 6-hydroxylase and isoflavone *O*-methyltransferase through a transcriptome analysis and related enzyme assays. Furthermore, using yeast engineered to express genes encoding these enzymes, we successfully produced the 6-methoxyisoflavones tectorigenin, glycitein, and afrormosin from the inexpensive isoflavones genistein, daidzein, and formononetin, respectively. The tectorigenin production rate was 40 mg l^−1^ after being cultured for 72 h. These results contribute to large-scale tectorigenin production and to tectorigenin-related research.

## Introduction

**Figure 1:**
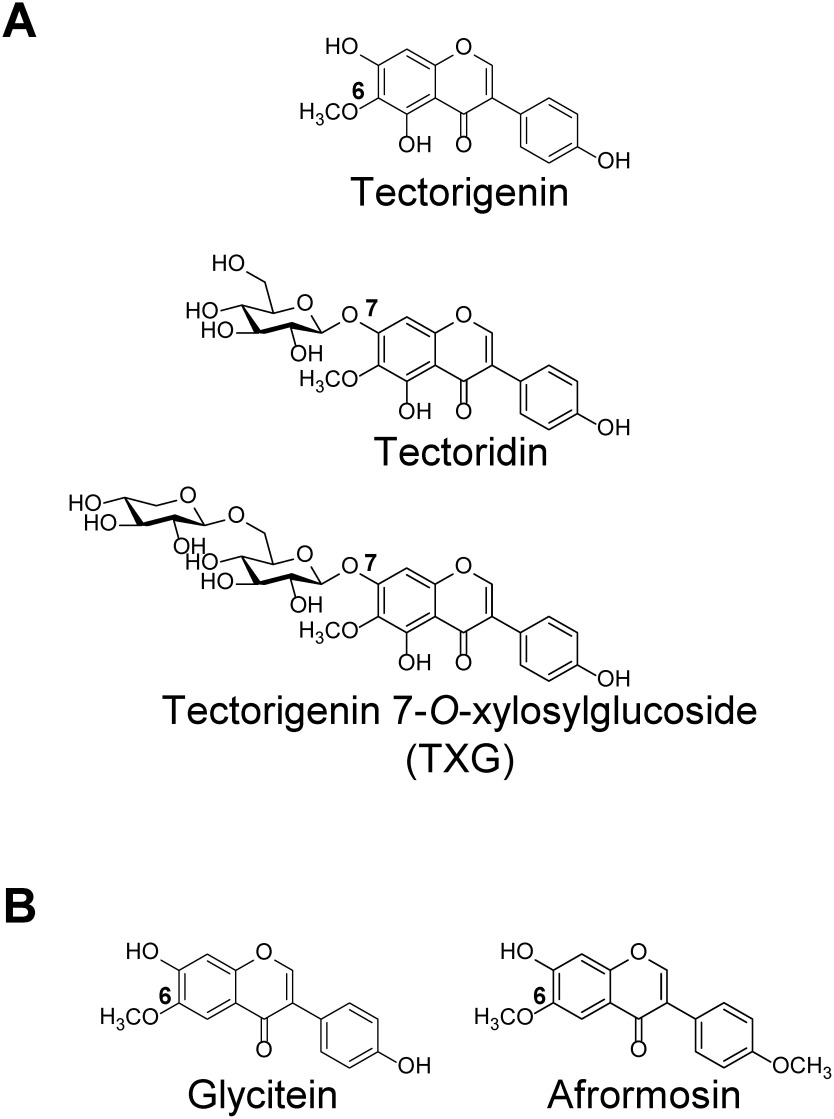
Figure 1. Isoflavones with a methoxylated 6-position. A, Tectorigenin derivatives detected in kudzu flower; B, Typical 6-methoxylated isoflavones identified in legumes.

Kudzu (*Pueraria montana* var. *lobata*) is a legume that accumulates isoflavones that have various beneficial effects for mammals, and its extracts are used as dietary supplements and in Chinese herbal medicine (Patil and Kumar 2025). 5,7,4′-Trihydroxy-6-methoxyisoflavone (tectorigenin), a type of isoflavone produced by members of the *Pueraria* genus, including kudzu, and its glycosides, tectorigenin 7-*O*-glucoside (tectoridin) and tectorigenin 7-*O*-xylosylglucoside (TXG) (Figure 1A), accumulate in flowers (Lee et al. 2000). Tectorigenin and/or its derivatives perform anti-cancer activities on human cells (Jiang et al. 2012; Yang et al. 2012; Zeng et al. 2018); therefore, they are important metabolites with the potential to contribute to human health.

Tectorigenin is a 6-methoxylated isoflavone produced by the hydroxylation of position 6, followed by methoxylation. To date, the following major three cytochrome P450 enzymes have been identified as flavonoid 6-hydroxylases (F6Hs), which hydroxylate position 6 of flavonoids: CYP82D1, involved in baicalein biosynthesis in *Scutellaria baicalensis* (Zhao et al. 2018); CYP706X1, involved in breviscapin biosynthesis in *Erigeron breviscapus* (Gao et al. 2022); and CYP71D9, involved in 7,4′-dihydroxy-6-methoxyisoflavone (glycitein) biosynthesis in soybean (*Glycine max*) (Latunde-Dada et al. 2001). While several F6Hs have been identified, only two isoflavone 6-hydroxylases have been identified to date: CYP76F17 and Medtr4g078110 from soybean and *Medicago truncatula*, respectively, which were very recently reported by Yang et al. (2025). However, these show activities against daidzein and formononetin, but not against genistein, which is the precursor of tectorigenin.

The major 6-methoxyisoflavones mostly produced by legumes are glycitein, tectorigenin, and 7-hydroxy-6,4′-dimethoxyisoflavone (afrormosin) (Dewick 1988) (Figure 1A, B). Of these, only the biosynthetic pathway of glycitein in soybean has been elucidated (Horitani et al. 2024; Latunde-Dada et al. 2001; Uchida et al. 2020). As described above, tectorigenin derivatives are useful compounds, but kudzu is not generally cultivated, and its flowers bloom only during a limited period, making large-scale production difficult and costly. Therefore, by elucidating the biosynthetic pathway of tectorigenin, its large-scale production using microbial conversion may be possible.

In this study, we performed a transcriptome analysis placing particular emphasis on kudzu floral tissues to elucidate the steps of tectorigenin biosynthesis, and using enzymatic assays, we identified isoflavone 6-hydroxylase (I6H), which hydroxylates position 6 of isoflavones. Furthermore, we identified isoflavone *O*-methyltransferase (IOMT), which has the activity of transferring a methyl group to position 6 of isoflavones, and we successfully produced approximately 40 mg l^−1^ of tectorigenin from genistein in a yeast expression system co-expressing I6H and IOMT. This research provides important insights into the production of rare 6-methoxyflavones, including tectorigenin.

## Materials and methods

### Chemicals and plant materials

Glycitein, 6-hydroxydaidzein, and 8-hydroxygenistein were purchased from Nagara Science Co., Ltd. (Gifu, Japan). Tectorigenin, tectoridin, daidzein, genistein, and formononetin were purchased from Tokyo Chemical Industry Co., Ltd. (Tokyo, Japan). Tectorigenin 7-*O*-xylosylglucoside, afrormosin, and 6-hydroxygenistein were purchased from TOKIWA PHYTOCHEMICAL (Chiba, Japan), INDOFINE Chemical Company, Inc. (Hillsborough, NJ, USA), and Biosynth (Gardner, MA, USA), respectively.

The organs of kudzu were sampled from wild plants at the authors’ institution in July and frozen using liquid nitrogen. Flower and bud samples were crushed using zirconia beads (5 mm) and a ShakeMaster NEO (1,000 rpm, 5 min; Bio Medical Science, Tokyo, Japan) with aluminum blocks cooled by liquid nitrogen. Leaf samples were crushed in liquid nitrogen using a mortar and pestle.

### Transcriptome analysis

Total RNA samples were extracted using ISOSPIN Plant RNA (Nippon Gene Co., Ltd., Tokyo, Japan). Library preparation using NEBNext Ultra II Directional RNA Library prep kit for Illumina (New England Biolabs Inc., Ipswich, MA, USA) and RNA-sequencing using Illumina NovaSeq 6000 (150 bp paired-end; San Diego, CA, USA) were outsourced to GENEWIZ from Azenta Life Sciences (South Plainfield, NJ, USA). The FASTQ files were registered in the DNA Data Bank of Japan Sequence Read Archive, and the transcriptome analysis was performed in accordance with previously reported methods (Uchida et al. 2025).

### Cloning

All the primer sequences used for cloning are listed in the Supplementary methods, and all the PCR amplifications were performed using PrimeSTAR Max DNA Polymerase (Takara, Shiga, Japan). cDNA was synthesized using total RNA from bud samples using the SuperScript IV First-Strand Synthesis System (Thermo Fisher Scientific, Waltham, MA, USA). The coding sequence of each candidate gene was amplified by PCR, introduced independently into pCR8/GW/TOPO (Thermo Fisher Scientific), and then, recombined into pYES-DEST52 (Thermo Fisher Scientific) using the Gateway LR Clonase II Enzyme mix (Thermo Fisher Scientific). *IOMT* was amplified and then assembled into the BamH1/Sal1 (RIKAKEN, Tokyo, Japan)-linearized pESC-LEU (Agilent Technologies, Santa Clara, CA, USA; Seki et al. 2008) including *Lotus japonicus* *cytochrome P450 reductase 1* (*LjCPR1*) using NEBuilder (New England Biolabs, Beverly, MA, USA).

### Assay of I6H in yeast

The yeast (*Saccharomyces cerevisiae*) BJ2168 strain was transformed with pYES-DEST52 (I6H) and pESC-LEU (LjCPR1) and then cultured on synthetic complete agar medium, without URA and LEU, at 30°C for 3 days. The growing colonies were incubated, with shaking at 200 rpm, overnight at 30°C in 10 ml of the same liquid medium. After collecting the cells by centrifugation (3,000 g, 5 min), they were resuspended in 10 ml of yeast peptone galactose liquid medium and divided into 5 ml aliquots. Then, 5 µl of substrate (50 µg µl^−1^ 1 : 1 dimethyl sulfoxide : ethanol) was added to each aliquot, and they were cultured under the same conditions. After 24 h of incubation, 200 µl of culture was extracted with 200 µl of ethyl acetate, and 150 µl of the ethyl acetate layer was dried using nitrogen gas. Each dried sample was dissolved in 200 µl of methanol and analyzed by liquid chromatography–photodiode array (LC–PDA). The analysis system was the same as previously reported by Uchida et al. (2021), and the gradient conditions were as follows: Solvent A, water with 0.1% formic acid; Solvent B, acetonitrile with 0.1% formic acid; and a linear gradient of Solvent B: 10–50% over 5 min. The same solvents were used in all the analyses.

### Quantification of tectorigenin derivatives in kudzu

The freeze-crushed samples were freeze-dried and dissolved in 80% (v/v) methanol containing 0.1% formic acid to achieve a concentration of 4 mg ml^−1^. The samples were then suspended at 1,000 rpm for 2 min using a ShakeMaster NEO (Bio Medical Science). After centrifugation, each supernatant was analyzed by LC–PDA with the following linear gradient of Solvent B: 10–50% over 5 min.

### Quantification of 6-methoxyisoflavones production

The amount of 6-methoxyisoflavone produced was calculated using yeast transformed with I6H (pYES-DEST52) and LjCPR1-IOMT (pESC-LEU) under the following slightly modified conditions: 10 µl of substrate was used; 100 µl of culture was extracted using 200 µl of ethyl acetate; 100 µl of the ethyl acetate layer was dried using nitrogen gas and dissolved in 200 µl of methanol; and the samples were analyzed by LC–PDA with the following linear gradient of Solvent B: 10–100% over 10 min.

## Results and discussion

### Analysis of tectorigenin derivatives in kudzu

**Figure 2:**
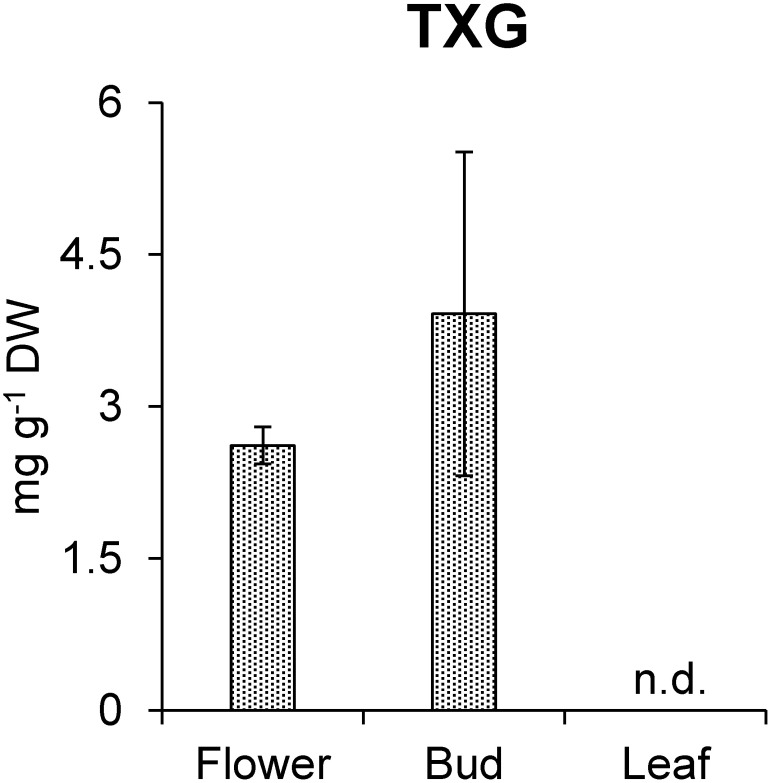
Figure 2. TXG content in each investigated kudzu organ. Error bars indicate means±standard errors (flower and bud, *n*=6; leaf, *n*=1). n.d., not detected; DW, dry weight.

Accumulations of tectorigenin derivatives have been reported in the flowers of *Pueraria* genus members (Lee et al. 2000). Consequently, we analyzed the kudzu samples for tectorigenin derivatives. Samples were extracted with 80% (v/v) methanol and analyzed using LC–PDA. As a result, only peaks having retention time and UV spectra matching those of TXG were detected in kudzu buds and flowers, whereas tectorigenin and tectoridin were not detected in any of the samples. The TXG level did not show a significant difference between flower and bud (Student *t*-test, *p* value=0.07) (Figure 2).

### Transcriptome analysis and identification of I6H

We performed RNA-sequencing using kudzu samples, constructed contigs by de novo assembly, and calculated expression levels as transcripts per million (TPM). In total, 345,851 contigs were generated (Supplementary Table S1).

**Figure 3:**
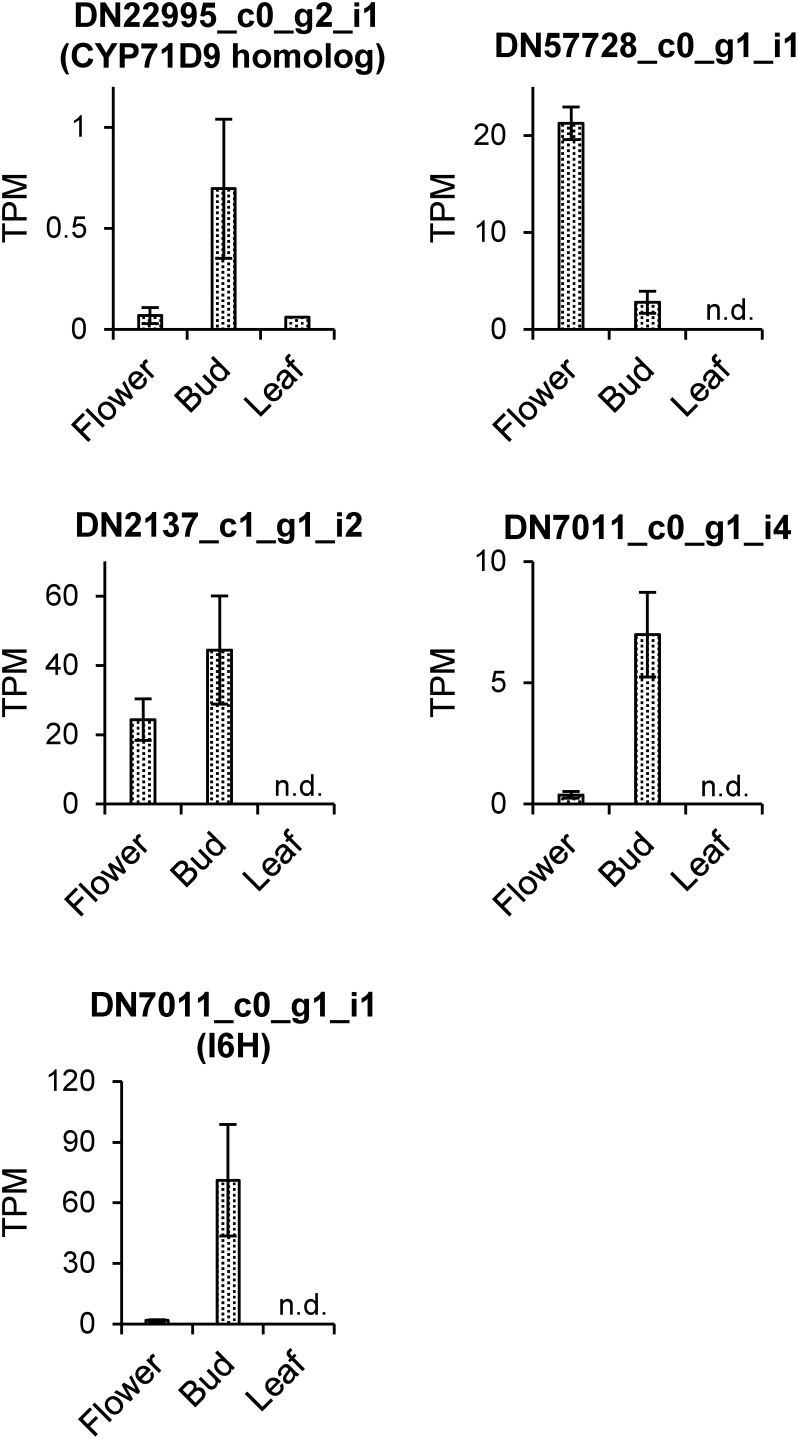
Figure 3. Expression levels of *I6H* candidate genes selected from the transcriptome analysis. Error bars indicate means±standard errors (flower and bud, *n*=6; leaf, *n*=1).

**Figure 4:**
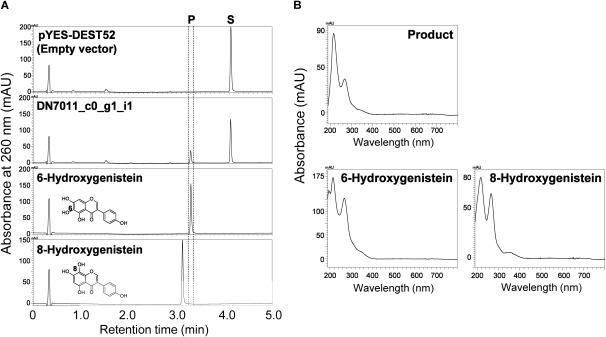
Figure 4. LC–PDA analysis of the *I6H* candidate gene assay. A, LC–PDA chromatogram of the ethyl acetate extract of the yeast culture medium. The chromatograms of 6- and 8-hydroxygenisteins are those of the reference standards; B, UV spectra of product and reference standard peaks; S, substrate (genistein); P, product.

Several cytochrome P450s have known 6- or 8- hydroxylase activities on flavonoids. Here, a BLASTP was performed using the amino acid sequence of soybean F6H (CYP71D9), which is involved in glycitein biosynthesis, as a query against the kudzu contigs, and a contig (DN22995_c0_g2_i1) showing a 76% amino acid identity was found. However, because the expression level of this contig was very low (TPM <1) in all the investigated organs (Figure 3), we concluded that it was not involved in tectorigenin biosynthesis, and it was not examined further. Next, we selected four cytochrome P450s from contigs that were expressed in buds or flowers, but not in leaves (Figure 3), and used these as candidate genes. Each candidate gene was introduced into yeast, and its protein expression was induced. Naringenin or genistein was added to each culture as a candidate substrate. After 24 h of incubation, the culture medium was extracted with ethyl acetate and analyzed using LC–PDA. A new peak was only confirmed for DN7011_c0_g1_i1 when genistein acted as the substrate (Figure 4A). The retention time and UV spectra of this peak matched those of the 6-hydroxygenistein, rather than 8-hydroxygenistein, standard (Figure 4A, B). Therefore, we concluded that DN7011_c0_g1_i1 encodes an I6H, rather than an F6H. Consequently, hydroxylation at position 6 during tectorigenin biosynthesis in kudzu flowers and buds was determined to occur at the isoflavone step rather than the flavanone step.

The identified I6H was annotated as belonging to the CYP76F subfamily using the P450 ATLAS (https://p450atlas.org/index.html) (Gront et al. 2025), and it was named CYP76F318 by Professor David R. Nelson (The University of Tennessee Health Science Center). A BLASTP search using the amino acid sequence of I6H as the query showed a high identity (81%) to CYP76F17 in soybean. Recently, CYP76F17 has been reported to affect the accumulation of glycitein in soybean seeds (Horitani et al. 2024), and I6H activities on daidzein and formononetin, but not genistein, have been identified (Yang et al. 2025). Therefore, kudzu-derived I6H was first discovered as an I6H having activity on genistein.

### Identification of IOMT in kudzu

**Figure 5:**
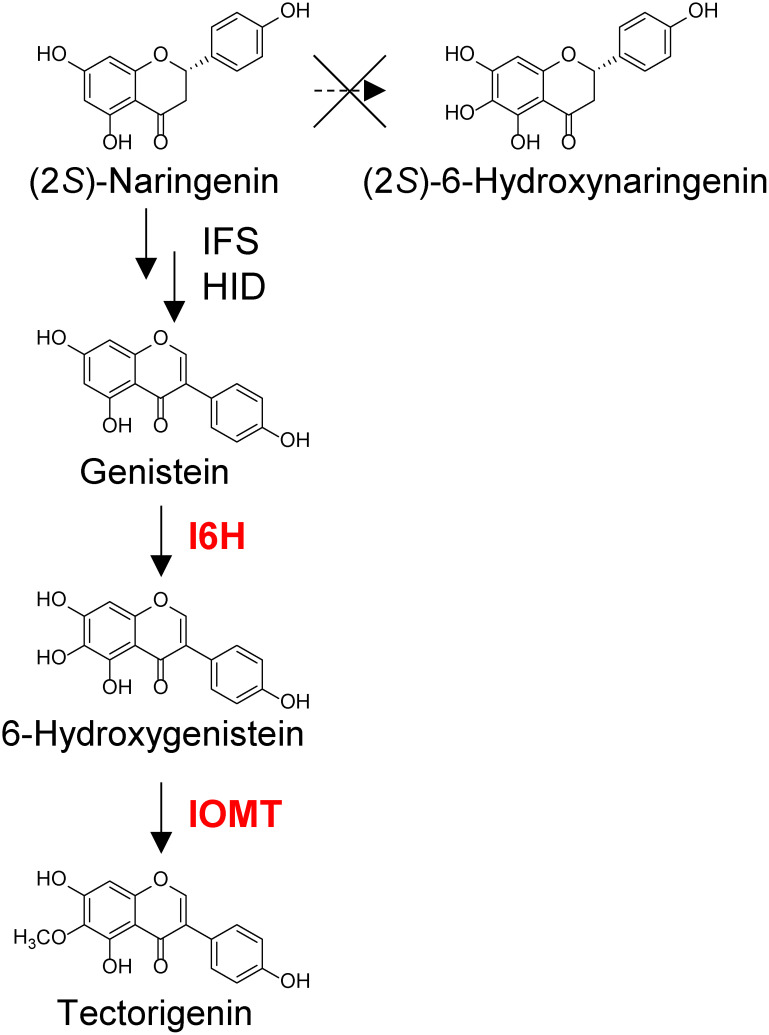
Figure 5. The biosynthetic pathway of tectorigenin in kudzu flowers. Hydroxylation at position 6 occurs at the genistein stage (an isoflavone) rather than at the naringenin stage (a flavanone), followed by methylation, in kudzu. IFS, 2-hydroxyisoflavanone synthase; HID, 2-hydroxyisoflavanone dehydratase.

Tectorigenin is an isoflavone with a methoxy group at position 6, and methylation is expected to occur after the formation of 6-hydroxygenistein by I6H. We previously reported that GmI6OMT1 is a cation-dependent OMT involved in soybean glycitein biosynthesis (Uchida et al. 2020), and consequently, we predicted that the OMTs involved in tectorigenin biosynthesis were also cation dependent. One *OMT* (DN4557_c0_g1_i5), which was significantly expressed in buds (Supplementary Figure S1), was found to have an expression pattern similar to that of *I6H* and was, therefore, selected as a candidate gene. I6H and this OMT were then co-expressed in yeast to confirm the production of tectorigenin using genistein as the starting material. A procedure similar to that used to identify I6H was performed, and the extract was analyzed using LC–PDA. The resulting peak was consistent with tectorigenin (Supplementary Figure S2), indicating that this OMT has IOMT activity on the 6 position. Thus, the enzymes involved in tectorigenin biosynthesis from genistein in kudzu flowers were elucidated (Figure 5).

In kudzu, TXG is detectable in both buds and flowers. In contrast, expression of the *I6H* and *IOMT* genes is strongly enriched in buds (Figure 3, Supplementary Figure S1). This pattern is consistent with a developmental progression in which these genes are transiently expressed at the bud stage, while the downstream metabolite accumulates during flower development.

### Production of 6-methoxyisoflavones in yeast

**Figure 6:**
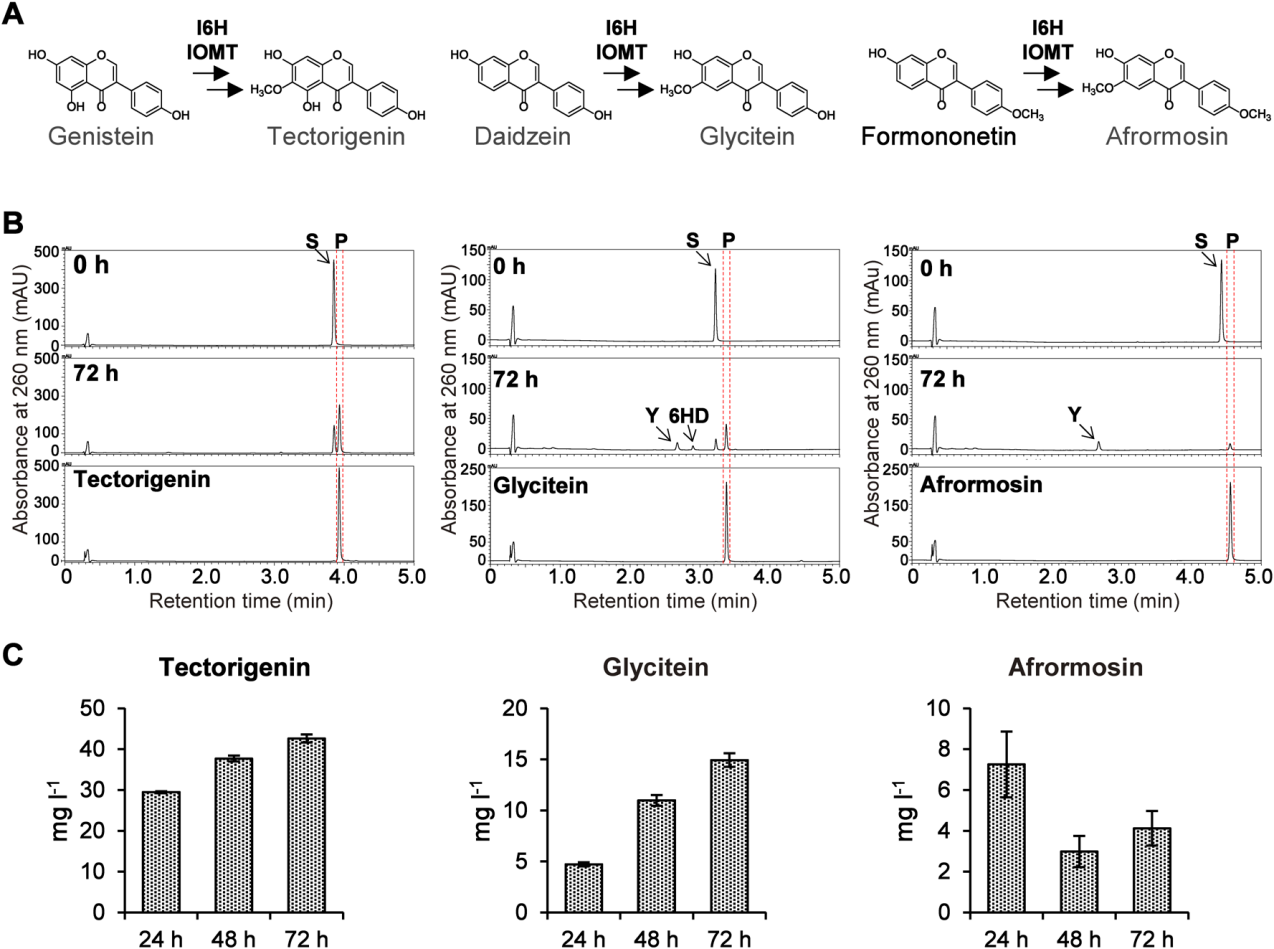
Figure 6. Production of 6-methoxylated isoflavones using I6H and IOMT. A, Substrates and final products of the bioconversion; B, Chromatograms of the culture medium at 0 h and 72 h after substrate addition and the standard. The peak enclosed by dotted lines indicates the final product; C, Bar graph quantifying the yields of bioconversion products. S, substrate (isoflavone); P, product (6-methoxyisoflavone); Y, yeast-derived impurities; 6HD, 6-hydroxydaidzein.

Genistein is an inexpensive isoflavone, whereas tectorigenin is approximately 60 times more expensive (refer to the price from Tokyo Chemical Industry Co., Ltd.). Because the co-expression of I6H and IOMT in yeast could produce tectorigenin from genistein, we attempted to produce expensive 6-methoxyisoflavones (tectorigenin, glycitein, and afrormosin) using inexpensive isoflavones (genistein, daidzein, and formononetin, respectively) as substrates (Figure 6A). The amount of each product was confirmed by sampling every 24 h for 72 h from the induction of protein expression. As a result, peaks of 6-methoxyisoflavones were detected in all the isoflavone-substrate experiments (Figure 6B). When using genistein, which was expected to be the original substrate of I6H in kudzu, the highest production rate was approximately 40 mg l^−1^ (Figure 6C). However, the maximum production rates of glycitein and afrormosin were approximately 15 and 7 mg l^−1^, respectively. In addition, the maximum production of afrormosin was observed after 24 h, and no correlation was observed between time and production level, suggesting degradation of the product or intermediates. Although there are reports of 6-hydroxyisoflavones being produced, to date, there have been no reports of them being produced from isoflavones. Previously, CYP57B3 from *Aspergillus oryzae* was expressed in *Pichia pastoris* to produce 6-hydroxydaidzein, with a yield of 9.1 mg l^−1^ (Chang et al. 2013; Nazir et al. 2011). Thus, it appears that kudzu I6H can produce 6-hydroxyisoflavones more efficiently than CYP57B3.

In this study, we identified I6H and IOMT and elucidated the biosynthesis of tectorigenin in kudzu. Furthermore, we demonstrated that these enzymes can be used to efficiently produce expensive 6-methoxyisoflavones from inexpensive isoflavones. Because 6-hydroxyisoflavones are either expensive or unavailable, limited research on their efficacies has been conducted. However, this study has demonstrated the feasibility of synthesizing 6-hydroxyisoflavones; therefore, further research into their various bioactivities is anticipated.

## Abbreviations

F6H: flavonoid 6-hydroxylase
I6H: isoflavone 6-hydroxylase
IOMT: isoflavone *O*-methyltransferase
I6OMT: isoflavone 6-*O*-methyltransferase
LC–PDA: liquid chromatography–photodiode array
LjCPR: *Lotus japonicus* cytochrome P450 reductase
TXG: tectorigenin 7-*O*-xylosylglucoside

## Accession numbers

RNA-sequencing, PRJDB35723 (DRR706751–DRR706764); I6H, LC886240; IOMT, LC886241.

## Description of supplementary files

Supplementary Methods. Primer list.

Supplementary Figure S1. Expression levels of *IOMT* candidate gene.

Supplementary Figure S2. In vivo genistein conversion in yeast through the co-expression of I6H and IOMT.

Supplementary Figure S3. Analysis of tectorigenin derivatives in kudzu.

Supplementary Table S1. List of TPM values for contigs obtained via RNA-sequencing.
