# Supplementary material for: Identification of an isoflavone 6-hydroxylase involved in tectorigenin biosynthesis in kudzu (Pueraria montana var. lobata) and the efficient production of 6-methoxyisoflavones
Source: Plant Biotechnol (Tokyo). 2026 Jun 25;43(2):193–9. doi: 10.5511/plantbiotechnology.26.0330b (PMC13324318; doi:10.5511/plantbiotechnology.26.0330b)
Supplement: Supplementary Data [file plantbiotechnology-43-2-26.0330b-s001.pdf]

Supplementary Methods. Primer list

Name	Forward (5′ > 3′)	Reverse (5′ > 3′)
DN57728_c0_g1_i1	ATGTACAACATTTCATTTCTCAATCTC	TTATTGATGGCCCAGTTTGGATGG
DN2137_c1_g1_i2	ATGGATCCTGCTTCATATGTCC	TCAAATGCTTTCATAAAGCTCCAATG
DN7011_c0_g1_i4	ATGGAGTTGCTAAGCTGTATAGTC	TTAGTGGCTGACTTTCTCGGC
DN7011_c0_g1_i1 (I6H)	ATGGAGTTTGTAAGTGTAAGCTGTG	TCATGACACGAGTTTCTCGGG
IOMT	AAAAAACCCCGGATCATGGCTAAGGAAGAGGAACAG	TCTGTTCCATGTCGATCAGATGATGCGGCGGCACATG

The underlining indicates sequences that are homologous to the plasmid.

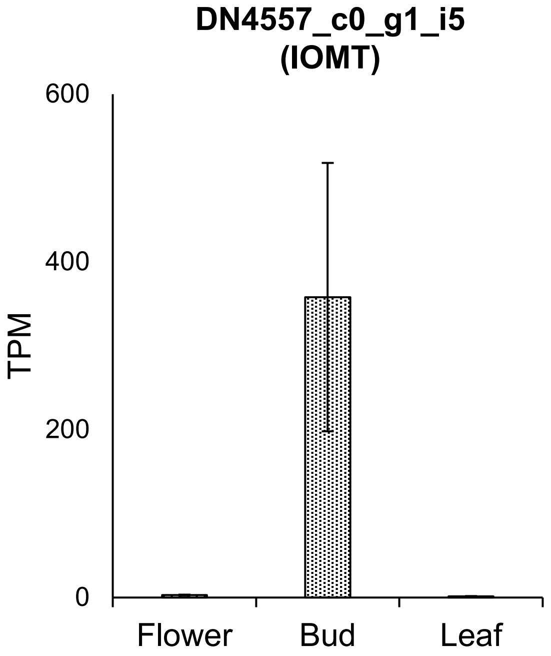

Supplementary Figure S1 Expression levels of *IOMT* candidate gene. Error bars indicate mean ± standard error.

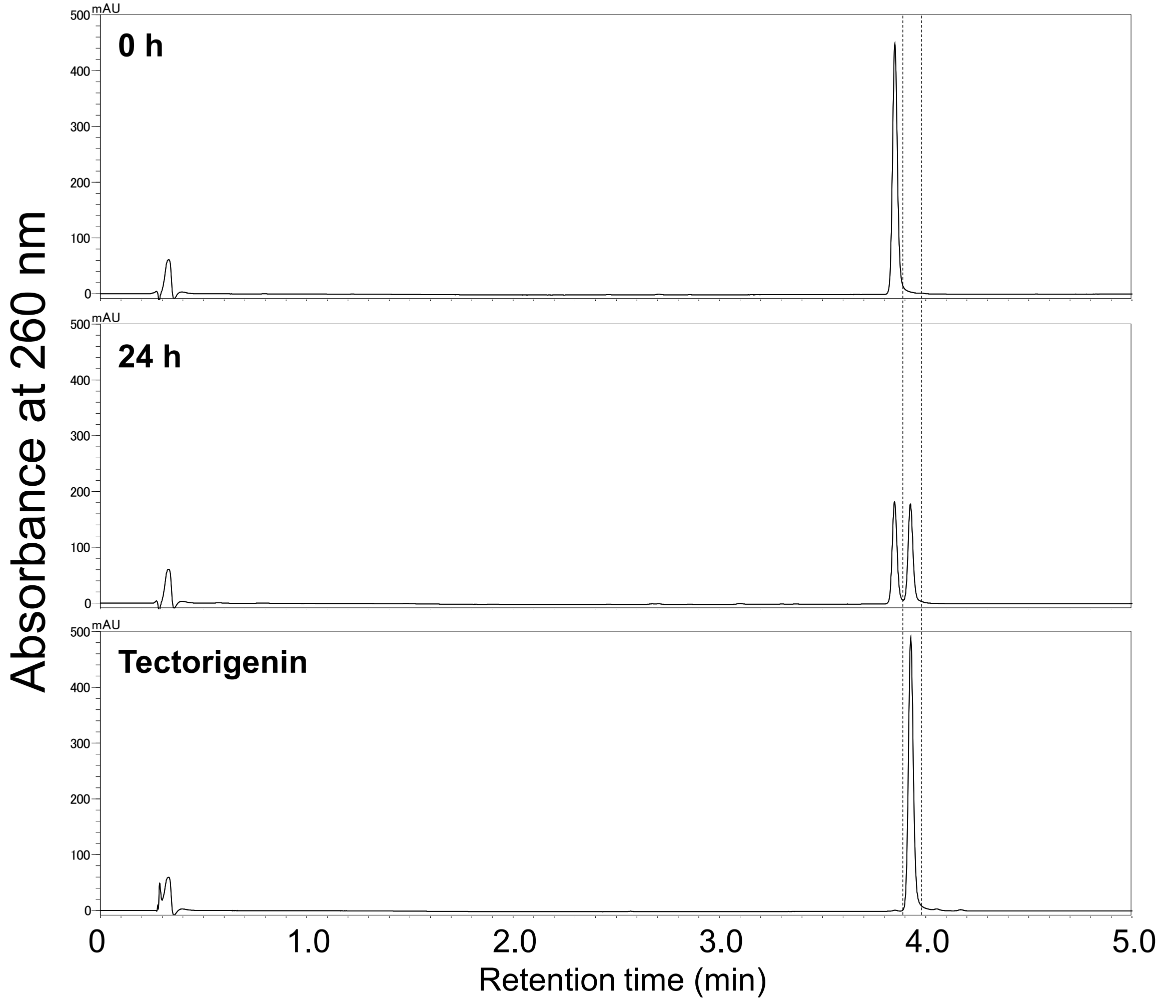

Supplementary Figure S2 In vivo genistein conversion in yeast through the co-expression of I6H and IOMT. The peak enclosed by dotted lines indicates the product.

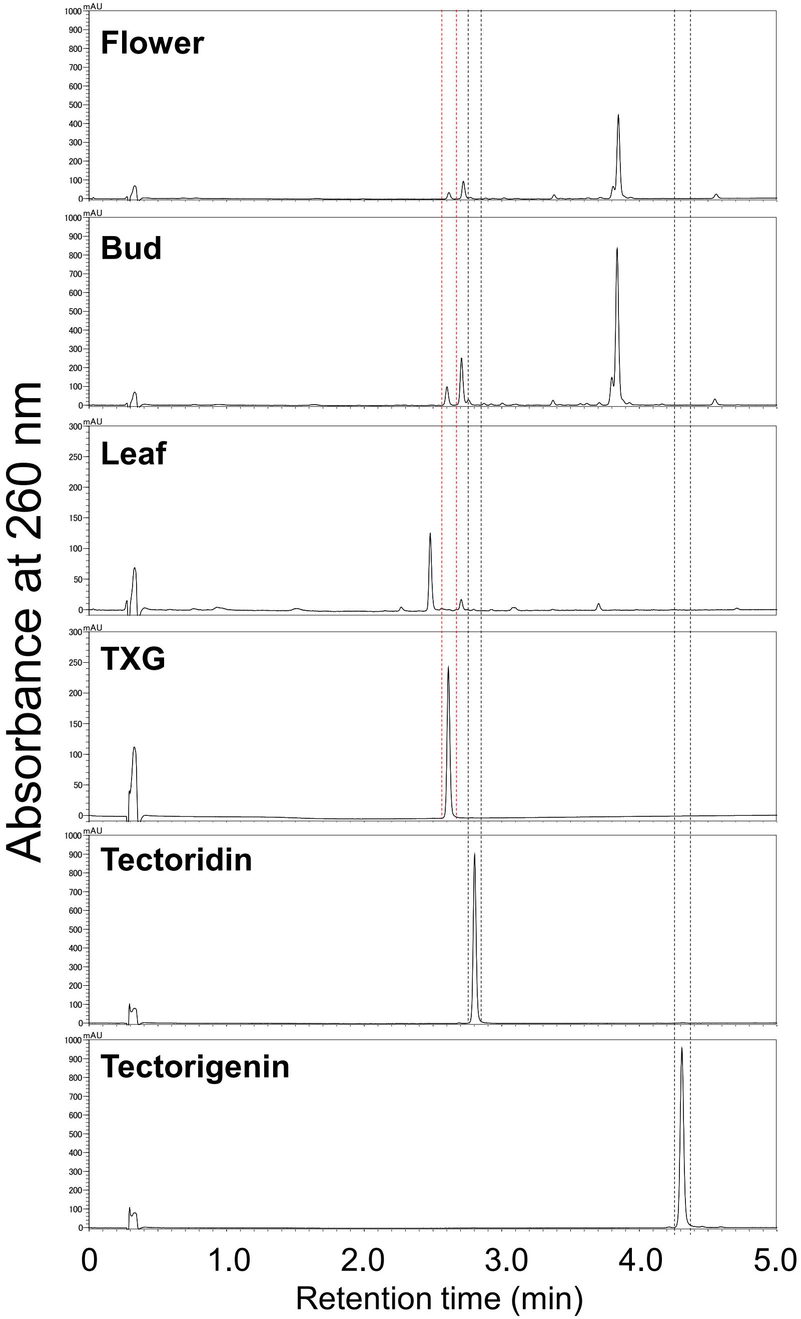

Supplementary Figure S3 Analysis of tectorigenin derivatives in kudzu.
The red dotted line indicates peaks with retention time and UV spectrum matching.
